# Acute Kidney Injury Among Children Likely Associated with Diethylene Glycol–Contaminated Medications — The Gambia, June–September 2022

**DOI:** 10.15585/mmwr.mm7209a1

**Published:** 2023-03-03

**Authors:** Parsa Bastani, Anna Jammeh, Frederica Lamar, Jason H. Malenfant, Peter Adewuyi, Alyson M. Cavanaugh, Kimberly Calloway, Carolyn Crisp, Nuha Fofana, T. Christy Hallett, Amadou Jallow, Uzoamaka Muoneke, Momodou Nyassi, Jerry Thomas, Alyssa Troeschel, Ellen Yard, Michael Yeh, Mustapha Bittaye

**Affiliations:** ^1^Epidemic Intelligence Service, CDC; ^2^Division of Global Health Protection, Center for Global Health, CDC; ^3^Ministry of Health, Banjul, The Gambia; ^4^Epidemiology Disease Control Unit, Ministry of Health, Banjul, The Gambia; ^5^Division of High-Consequence Pathogens and Pathology, National Center for Emerging and Zoonotic Infectious Diseases, CDC; ^6^The Gambian Field Epidemiology Training Program, Banjul, The Gambia; ^7^African Field Epidemiology Network, Kampala, Uganda; ^8^Division of Foodborne, Waterborne, and Environmental Diseases, National Center for Emerging and Zoonotic Infectious Diseases, CDC; ^9^Division of Environmental Health Science and Practice, National Center for Environmental Health, CDC; ^10^Ghana Field Epidemiology and Laboratory Training Program, Accra, Ghana; ^11^Department of Pediatrics, University of Nigeria, Nsukka, Enugu, Nigeria; ^12^Department of Pediatrics, Edward Francis Small Teaching Hospital, Banjul, The Gambia; ^13^Division of Laboratory Sciences, National Center for Environmental Health, CDC.

On July 26, 2022, a pediatric nephrologist alerted The Gambia’s Ministry of Health (MoH) to a cluster of cases of acute kidney injury (AKI) among young children at the country’s sole teaching hospital, and on August 23, 2022, MoH requested assistance from CDC. CDC epidemiologists arrived in The Gambia, a West African country, on September 16 to assist MoH in characterizing the illness, describing the epidemiology, and identifying potential causal factors and their sources. Investigators reviewed medical records and interviewed caregivers to characterize patients’ symptoms and identify exposures. The preliminary investigation suggested that various contaminated syrup-based children’s medications contributed to the AKI outbreak. During the investigation, MoH recalled implicated medications from a single international manufacturer. Continued efforts to strengthen pharmaceutical quality control and event-based public health surveillance are needed to help prevent future medication-related outbreaks.

## Investigation and Results

In July 2022, a pediatric nephrologist at the Edward Francis Small Teaching Hospital in The Gambia’s capital city of Banjul alerted MoH to a cluster of AKI cases among children. Data on AKI incidence are not routinely collected in The Gambia; thus, the baseline AKI rate was unknown. However, the treating nephrologist expressed concern that the number of cases and deaths were well above baseline: through August 12, a total of 30 pediatric deaths among 37 AKI cases had been reported from facilities throughout the country (case fatality rate = 81%). In response to a request for assistance, a multidisciplinary CDC team, including an epidemiologist, an anthropologist, an infectious disease physician, and an environmental health scientist, arrived on September 16 to assist the Field Epidemiology Training Program (FETP) and MoH in characterizing the illness and identifying exposures. MoH collaborated separately with the World Health Organization (WHO) to test medications that might have been used by patients.

Active surveillance was conducted through reoccurring communication with the teaching hospital and other regional hospitals, inquiring about admissions of pediatric patients with kidney failure. As of September 29, 2022, MoH had identified 78 clinically suspected AKI cases. Among these patients, 66 (85%) had died. Most patients (75%) were aged <2 years, and 60% were male. Cases were reported from six of the country’s seven health regions.* Among the 78 cases reported to MoH, symptom onset date was available for 67 (86%) (Figure).

**FIGURE:**
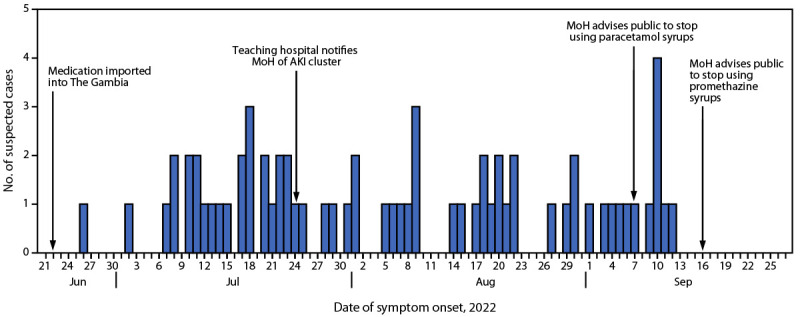
Date of first symptom onset for suspected cases of acute kidney injury of unknown etiology* among children (N = 67),^†^ critical events in the outbreak investigation, and public health recommendations — The Gambia, June 12–September 29, 2022 **Abbreviations**: AKI = acute kidney injury; MoH = The Gambia’s Ministry of Health. * Baseline incidence of AKI not available. These data include cases from MoH Epidemiology and Disease Control Unit with reported date of initial symptom onset. ^†^ Among 78 cases reported to MoH, symptom onset date was available for 67 (86%) patients.

CDC, MoH, and FETP investigators developed a standardized case report form to gather data about pediatric patients with AKI identified by MoH. Data collection consisted of a review of the patient’s medical records and caregiver interview data. For this investigation, a confirmed case of pediatric AKI was defined as anuria (no urine output) of unknown etiology in a child aged ≤8 years, persisting for ≥24 hours, during June 21–September 29, 2022. June 21 was chosen as the start date because medications suspected to have possibly caused the AKI outbreak had been imported into the country on June 21. Investigators first reviewed the medical records of 52 (67%) of the 78 MoH-identified patients with suspected AKI who were treated by the teaching hospital. Next, interviews with at least one caregiver of each of 27 patients were conducted by investigators in local languages, including the caregivers of 20 patients whose children’s hospital medical records were reviewed and seven patients whose medical records could not be located. Investigators selected a convenience sample of caregivers for interview based on their location of residence. Because of resource limitations and reluctance on the part of some caregivers to be interviewed, not all medical records could be located, and not all invited caregivers participated. In addition, interview data were collected in only two of the six health regions (Western Regions 1 and 2). This activity was reviewed by CDC and was conducted consistent with applicable federal law and CDC policy.^†^

Caregivers were asked about the child’s recent medical history, including disease course, experiences with the health care system, and possible exposures, including medications, foods, and experience of flooding, among others. All participating caregivers were interviewed in their homes. All four (15%) living patients whose caregivers were interviewed had been discharged at the time of interview. Among patients who had died, the mean interval between date of death and date caregivers were contacted for interview was 33 days. Information from medical records and interviews was used to determine whether patients on the MoH line list met the confirmed case definition. After data from the 27 caregiver interviews and 52 medical record reviews were combined, 59 case report forms were completed, representing 76% of the 78 cases reported to MoH, and 88% of the 67 patients with a symptom onset date. Among these 59 patients, 56 (95%) met the confirmed case definition and were included in the analysis.^§^

Among the 56 included patients, more than one half (54%) experienced fever as their first signs or symptoms, and 50% experienced vomiting (Table 1). Approximately one third (34%) had diarrhea or loose stool as the first symptom. During the course of illness, all patients experienced anuria and fever, 95% experienced vomiting, and 73% experienced diarrhea. Nearly one half (48%) experienced anorexia or reduced feeding. The median interval from symptom onset to anuria was 5 days (IQR = 2–7 days); among the 26 patients who died, the median interval from onset of anuria to death was 6 days (IQR = 3–7 days).

**TABLE 1:** Signs and symptoms reported at the onset of illness and at any time during illness, intervals between onset of symptoms and onset of anuria, intervals between onset of anuria and death, and available medical records or caregiver interviews among pediatric patients with acute kidney injury (N = 56)* — The Gambia, June–September 2022

Symptoms and intervals until anuria or death	No. (%)^†^	No. of valid responses (median, [IQR])
**First symptoms experienced**		
Fever	30 (54)	NA
Vomiting	28 (50)	NA
Diarrhea/Loose stool	19 (34)	NA
Cough, runny nose, sneezing or unspecified cold symptom	7 (13)	NA
Anuria	2 (4)	NA
Anorexia	2 (4)	NA
Other^§^	5 (9)	NA
**Symptoms ever experienced**		
Anuria	56 (100)	NA
Fever	56 (100)	NA
Vomiting	53 (95)	NA
Diarrhea/Loose stool	41 (73)	NA
Reduced appetite/Decreased breastfeeding	27 (48)	NA
Difficulty breathing	22 (39)	NA
Cough, runny nose, sneezing or unspecified cold symptom	19 (34)	NA
Rash	6 (11)	NA
Abdominal pain	5 (9)	NA
Convulsions/Seizures	4 (7)	NA
Bloody diarrhea	1 (2)	NA
Jaundice	0 (—)	NA
**Intervals from symptom onset to anuria (or death)** ^¶^		
First symptoms experienced to anuria, days	NA	31 (5 [2 to 7])
Vomiting onset to anuria, days	NA	25 (2 [1 to 6])
Loss of appetite/Refusal to breastfeed onset to anuria, days	NA	17 (1 [0 to 1])
Diarrhea onset to anuria, days	NA	17 (4 [2 to 6])
Difficulty breathing onset to anuria, days	NA	7 (0 [–3 to 1]**)
Onset of anuria to death, days	NA	26 (5.5 [3 to 7])

**Abbreviation**: NA = not available.

* A confirmed case of pediatric acute kidney injury was defined as anuria (no urine output) of unknown etiology in a child aged ≤8 years, persisting for ≥24 hours, during June 21–September 29, 2022.

^†^ Percentages might sum to >100%, because multiple signs or symptoms could be reported for each patient.

^§^ Other reported symptoms included rash (one), headache (one), mouth ulcers (one), abdominal pain (one), and reduced urine output (one).

^¶^ Responses were considered valid if patients had the signs or symptoms of interest and had onset dates for both variables involved in the interval. For example, the interval vomiting onset to anuria was only calculated among patients who experienced vomiting and anuria and had recorded onset dates available for both symptoms.

** A negative value for this interval indicates that anuria preceded breathing difficulties.

Abnormal laboratory test results were received by 66%–100% of patients, including impaired renal and liver function, thrombocytosis, and mild to moderate anemia (Table 2). Fourteen patients underwent peritoneal dialysis, and one patient underwent hemodialysis; all 15 patients who received dialysis died.

**TABLE 2:** Laboratory test results among patients with acute kidney injury following medication exposures — Edward Francis Small Teaching Hospital, The Gambia, June–September 2022

Laboratory test	Normal reference range	Median patient value (IQR)	Abnormal test results	
			No./Total no. (%)	Direction of abnormality
Hemoglobin	11.5–16.5 g/dL	10.3 (9.0–11.5)	31/38 (82)	Low
Alanine transaminase	0–41 U/L	284.5 (135.0–473.0)	13/18 (72)	High
Alkaline phosphatase	45–135 U/L	272.5 (212.0–350.0)	14/14 (100)	High
Aspartate transferase	0–40 U/L	271.3 (176.5–610.5)	13/16 (81)	High
Blood urea nitrogen	1.7–8.3 mmol/L	29.8 (16.0–41.0)	37/41 (90)	High
Creatinine	44–123 *μ*mol/L	711.0 (499.5–854.0)	34/38 (89)	High
Platelets	130–400 x 10^9^/L	511.0 (388.0–598.0)	23/35 (66)	High

Among the 26 patients with a caregiver interview, 100% of caregivers reported that the child consumed a prescription or over-the-counter syrup-based medication (including paracetamol, known in the United States as acetaminophen, commonly administered for fever) before the onset of anuria. Twelve patients (47%) had consumed four or more medications before being hospitalized. Although many caregivers were unable to recall the names of medications that they administered to their children, caregivers of 14 (54%) of these 26 patients identified the manufacturer name of at least one medication administered to their child before inpatient hospitalization. A single international manufacturer that produced a syrup-based medication was reported in eight of 14 (57%) interviews in which caregivers identified the manufacturer name of at least one medication administered to their child before inpatient hospitalization. 

## Public Health Response

Preliminary reports from the treating physicians at the teaching hospital indicated that caregivers had administered paracetamol and promethazine to their children before their development of AKI. Based on this information, MoH advised the public to suspend use of all paracetamol and promethazine syrups on September 7, 2022, and September 16, 2022, respectively (Figure). The laboratory analysis of 23 medication samples conducted by MoH and WHO confirmed that four products from Maiden Pharmaceuticals Limited (Haryana, India) contained diethylene glycol (DEG) and ethylene glycol (EG). Based on records from The Gambia’s Medicines Control Agency, all medications that tested positive for DEG and EG were imported into The Gambia on June 21, 2022, shortly before the occurrence of the first AKI cases.

On October 4, 2022, MoH suspended the importation of all medications from this manufacturer and requested that health care providers stop prescribing, dispensing, and using medications produced by this manufacturer because of their possible contamination. On October 5, 2022, WHO issued a worldwide medical product alert for four syrup-based medications from Maiden Pharmaceuticals (*1*). In collaboration with WHO, UNICEF, ChildFund The Gambia, and The Gambia Red Cross Society, MoH supported a house-to-house recall and collection of all products from this manufacturer in addition to all paracetamol, promethazine, and cough syrups. In conjunction with the recall, MoH promoted awareness about the contaminated medications through messaging on radio, television, social media, and in places of worship. MoH also commenced ongoing pharmacy spot checks to ensure that products from this manufacturer were not being sold. Surveillance officers were trained in case definitions, case report forms, following up with patients discharged from hospitals, and conducting active case finding in the community.

## Discussion

This investigation strongly suggests that medications contaminated with DEG or EG imported into The Gambia led to this AKI cluster among children. AKI outbreaks associated with DEG-contaminated pharmaceutical products have been documented in Panama, Nigeria, India, and Haiti (*2**–**5*). Patients with DEG poisoning can experience a range of signs and symptoms, including altered mental status, headache, and gastrointestinal symptoms; however, the most consistent manifestation is AKI, characterized by oliguria (low urine output) or anuria, progressing over 1–3 days to renal failure (indicated by elevated serum creatinine and blood urea nitrogen) (*6*). In past DEG outbreaks, manufacturers have been suspected of substituting DEG in the place of more expensive, pharmaceutical-grade solvents (*3*,*7*). Medication testing supported contaminated medical syrups as the etiology of this cluster of AKI cases (*1*).

Further support for a toxic etiology includes the wide geographic distribution of cases in the country (six of seven health regions), a common pharmaceutical manufacturer of medications reported to have been used by many patients, and a low rate of intrahousehold spread. This intoxication appears to have only affected children, likely because medications in syrup form are most commonly used for children in The Gambia.

Factors that might have led to poor caregiver recall regarding medications administered to their children include the assumption by caregivers that medications would not harm their children, difficulty of parents of children who had died recalling administered medications owing to the interval between patients’ deaths and the investigation, and the possibility of reduced reporting following the issuing of health alerts by MoH.

This likely poisoning event highlights the potential public health risks posed by the inadequate quality management of pharmaceutical exports. Among reports of AKI associated with DEG-contaminated medical products, this is the first in which DEG-contaminated medications were imported into a country, rather than being domestically manufactured. Inadequate regulatory structures make the sale of medications from international markets an especially high-risk activity in low-resource settings. Medications for export might be subject to less rigorous regulatory standards than those for domestic use (*8*). Simultaneously, low-resource countries might not have the human and financial resources to monitor and test imported drugs (*8*). In addition to improving pharmaceutical quality management, efforts should be made by MoH to strengthen event-based surveillance systems (*9*), which rely on unstructured information (e.g., reports, rumors, and other information) to detect unusual events that might signal an outbreak or other public health event. Event-based surveillance complements traditional reportable disease surveillance and has the potential to improve the ability to rapidly detect and respond to similar acute health events of unknown and unexpected etiologies. FETP-trained experts can provide valuable assistance in these and other efforts to help governments improve their capacities to detect and respond to outbreaks in the future.

**Summary**

**What is already known about this topic?**

Diethylene glycol (DEG)–contaminated medications present a global health threat, especially in low-income countries.

**What is added by this report?**

A large cluster of acute kidney injury cases affecting children in The Gambia in 2022 was associated with case fatality rates >80%. The implicated syrup-based pediatric medications that had been administered to patients were imported from a single Indian manufacturer. This is one of the first documented DEG outbreaks in which contaminated medications were imported rather than being domestically manufactured.

**What are the implications for public health practice?**

DEG mass poisonings continue to occur worldwide, especially in low-resource settings. Strengthened international pharmaceutical regulatory structures and event-based surveillance systems can help prevent DEG-associated large-scale poisoning events.

## Footnotes

* Cases were reported from Western Region 1, Western Region 2, Upper River Region, North Bank West Region, Lower River Region, and Central River Region. Cases were not reported from the North Bank East Region.

† 45 C.F.R. part 46, 21 C.F.R. part 56; 42 U.S.C. Sect. 241(d); 5 U.S.C. Sect. 552a; 44 U.S.C. Sect. 3501 et seq.

§ The three patients who did not meet the case definition did not have documentation of anuria.

## References

[1] World Health Organization. Substandard (contaminated) pediatric medicines identified in WHO region of Africa. Geneva, Switzerland: World Health Organization; 2022. Accessed November 11, 2022. https://www.who.int/news/item/05-10-2022-medical-product-alert-n-6-2022-substandard-(contaminated)-paediatric-medicines

[2] Rentz ED, Lewis L, Mujica OJ, Outbreak of acute renal failure in Panama in 2006: a case-control study. Bull World Health Organ 2008;86:749–56. 10.2471/BLT.07.049965 18949211 PMC2649516

[3] CDC. Fatal poisoning among young children from diethylene glycol-contaminated acetaminophen—Nigeria, 2008–2009. MMWR Morb Mortal Wkly Rep 2009;58:1345–7. 20010509

[4] Singh J, Dutta AK, Khare S, Diethylene glycol poisoning in Gurgaon, India, 1998. Bull World Health Organ 2001;79:88–95. 11242827 PMC2566350

[5] CDC. Fatalities associated with ingestion of diethylene glycol-contaminated glycerin used to manufacture acetaminophen syrup—Haiti, November 1995–June 1996. MMWR Morb Mortal Wkly Rep 1996;45:649–50. 8769471

[6] Schep LJ, Slaughter RJ, Temple WA, Beasley DMG. Diethylene glycol poisoning. Clin Toxicol (Phila) 2009;47:525–35. 10.1080/15563650903086444 19586352

[7] Schier JG, Rubin CS, Miller D, Barr D, McGeehin MA. Medication-associated diethylene glycol mass poisoning: a review and discussion on the origin of contamination. J Public Health Policy 2009;30:127–43. 10.1057/jphp.2009.2 19597445

[8] Caudron JM, Ford N, Henkens M, Macé C, Kiddle-Monroe R, Pinel J. Substandard medicines in resource-poor settings: a problem that can no longer be ignored. Trop Med Int Health 2008;13:1062–72. 10.1111/j.1365-3156.2008.02106.x 18631318

[9] Balajee SA, Salyer SJ, Greene-Cramer B, Sadek M, Mounts AW. The practice of event-based surveillance: concept and methods. Glob Secur Health Sci Policy 2021;6:1–9. 10.1080/23779497.2020.1848444

